# Improvement of ablative margins by the intraoperative use of CEUS-CT/MR image fusion in hepatocellular carcinoma

**DOI:** 10.1186/s12885-016-2306-1

**Published:** 2016-04-18

**Authors:** Kai Li, Zhong-Zhen Su, Er-Jiao Xu, Jin-Xiu Ju, Xiao-Chun Meng, Rong-Qin Zheng

**Affiliations:** Department of Medical Ultrasonics, Third Affiliated Hospital of Sun Yat-Sen University, 600 Tianhe Road, Guangzhou, 510630, Guangdong Province PR China; Department of Radiology, Third Affiliated Hospital of Sun Yat-Sen University, 600 Tianhe Road, Guangzhou, 510630, Guangdong Province PR China

**Keywords:** Hepatocellular carcinoma, Ablative margin, Image fusion, Intraoperative, Contrast-enhanced ultrasound

## Abstract

**Background:**

To assess whether intraoperative use of contrast-enhanced ultrasound (CEUS)-CT/MR image fusion can accurately evaluate ablative margin (AM) and guide supplementary ablation to improve AM after hepatocellular carcinoma (HCC) ablation.

**Methods:**

Ninety-eight patients with 126 HCCs designated to undergo thermal ablation treatment were enrolled in this prospective study. CEUS-CT/MR image fusion was performed intraoperatively to evaluate whether 5-mm AM was covered by the ablative area. If possible, supplementary ablation was applied at the site of inadequate AM. The CEUS image quality, the time used for CEUS-CT/MR image fusion and the success rate of image fusion were recorded. Local tumor progression (LTP) was observed during follow-up. Clinical factors including AM were examined to identify risk factors for LTP.

**Results:**

The success rate of image fusion was 96.2 % (126/131), and the duration required for image fusion was 4.9 ± 2.0 (3–13) min. The CEUS image quality was good in 36.1 % (53/147) and medium in 63.9 % (94/147) of the cases. By supplementary ablation, 21.8 % (12/55) of lesions with inadequate AMs became adequate AMs. During follow-up, there were 5 LTPs in lesions with inadequate AMs and 1 LTP in lesions with adequate AMs. Multivariate analysis showed that AM was the only independent risk factor for LTP (hazard ratio, 9.167; 95 % confidence interval, 1.070–78.571; *p* = 0.043).

**Conclusion:**

CEUS-CT/MR image fusion is feasible for intraoperative use and can serve as an accurate method to evaluate AMs and guide supplementary ablation to lower inadequate AMs.

## Background

Percutaneous ablation is one of the most frequently used methods for hepatocellular carcinomas (HCCs) that are not suitable for resection or liver transplantation. Compared with resection, percutaneous radiofrequency ablation (RFA) has a higher rate of local tumor progression (LTP) [1–3], and the LTP rate contributes to long-term survival [4]. The ablative margin (AM) is an independent factor affecting LTP [5, 6]. Several studies have reported different methods to evaluate AM, including MR with impaired clearance of ferucarbotran [7, 8], MR with gadolinium ethoxybenzyl diethylene triamine pentaacetic acid [9], CT-CT image fusion [10, 11], MR-MR image fusion [12] and ultrasound-CT/MR image fusion [13]. In these studies, none of the methods were applied intraoperatively. Theoretically, if the AM could be assessed intraoperatively, supplementary ablation could be performed to increase the number of adequate AMs and reduce the probability of LTP.

Ultrasound has the advantages of real-time guidance, accessibility and non-invasiveness, and contrast-enhanced ultrasound (CEUS) has greatly improved the accuracy of ultrasound in liver tumor diagnosis and the evaluation of local ablation treatment [14]. Compared with CT/MR and the new imaging methods mentioned above, CEUS is more suitable for intraoperative usage. However, the sensitivity of CEUS within 1 h after ablation has been variable in many different studies [15–17] and can be as low as 25 % [17] due to interference by peripheral hyperemia or gas in the ablative area. Furthermore, AMs can not be accurately evaluated by routine CEUS.

Ultrasound-CT/MR image fusion combines the advantages of ultrasound and CT/MR and expands the use of both imaging methods, including the localization, identification and ablation of lesions that are not visible with B-mode ultrasound in the liver [18–21] as well as prostate gland [22]. The AM can be accurately evaluated by a precise comparison of the size and location of the tumor with the ablative area using CEUS and CT/MR image fusion one month after ablation [13]. However, whether CEUS-CT/MR image fusion can be applied intraoperatively to evaluate AM has not been reported. We hypothesized that if we combined the CEUS of the ablative area with the CT/MR image of the HCC before ablation, the AM could be evaluated intraoperatively. If the location of the inadequate AM could be identified, supplementary ablation could be performed. The aim of this study was to assess whether CEUS-CT/MR image fusion could be applied intraoperatively to evaluate the AM and guide supplementary ablation to facilitate the achievement of adequate AMs and, accordingly, reduce the rate of LTP.

## Methods

This prospective study was approved by The Institute Research Medical Ethics Committee of the Third Affiliated Hospital of Sun Yat-Sen University. All human studies were performed in accordance with the ethical standards established by the 1964 Declaration of Helsinki and its subsequent amendments. Informed consent was obtained from all patients prior to their inclusion in the study.

### Patients and lesions

The patients in this study were part of the ultrasound-CT/MR image fusion research program and were continuously enrolled every other week. From September 2009 to June 2012, all patients were enrolled who were diagnosed with HCC and scheduled to receive percutaneous ablation treatment in our department. All lesions were diagnosed based on the clinical criteria from the American Association for the Study of Liver Diseases [23]. Patients were excluded from the study if they met the following criteria: 1) patient was scheduled to receive other surgeries along with RFA that might affect ultrasound-CT/MR image fusion, such as liver resection, laparoscopic cholecystectomy or splenectomy; 2) ultrasound and CT/MR images could not be successfully fused; 3) patient was allergic to the ultrasound contrast agent; or 4) patient did not receive a CT/MR examination 1–2 months after ablation.

### RFA procedure

The Cool-Tip Radiofrequency System (Covidien, Mansfield, MA, USA) and single electrode with 3 cm long exposed tip were used. The ablation was performed under endotracheal anesthesia. Patients with lesions larger than 3 cm in diameter and/or with multiple lesions would receive transcatheter arterial chemoembolization (TACE) 1–2 weeks before RFA. All lesions were ablated according to a previously determined plan, and effort was made to ablate the whole tumor as well as the 5-mm AM. If the lesion was located within 5 mm of a blood vessel (portal vein or hepatic vein) less than 2 mm in diameter, effort would be made to ablate the lesion as well as the vessel to achieve an adequate AM. If the lesion was within 5 mm of a blood vessel larger than 2 mm in diameter, we attempted to ablate the entire normal hepatic parenchyma between the lesion and the vessel. If the HCC lesion was within 5 mm of critical structures such as the diaphragm or gastrointestinal tract, artificial ascites or pleural effusion was applied by injecting normal saline into the abdominal or pleural cavity to avoid injury to the critical structures. Supplementary ablation was applied if an adequate AM (5 mm) was not achieved after the previously planned ablation. Supplementary ablation was not performed if the inadequate AM was caused by large vessels (diameter >2 mm) or supplementary puncture was too risky or difficult.

### Evaluation of the AM by intraoperative CEUS-CT/MR image fusion

The MyLab Twice (Esaote, Italy) ultrasound unit and convex array transducer CA431 (4–10 MHz) were used. The ultrasound unit was equipped with the program Virtual Navigator, and the real-time contrast-tuned imaging technique (CnTI, MI < 0.05). SonoVue (Bracco, Italy) was used as the contrast agent. During each application, 2.4 ml of the contrast agent was administered into the antecubital vein followed by 5 ml of normal saline.

**Fig. 1:**
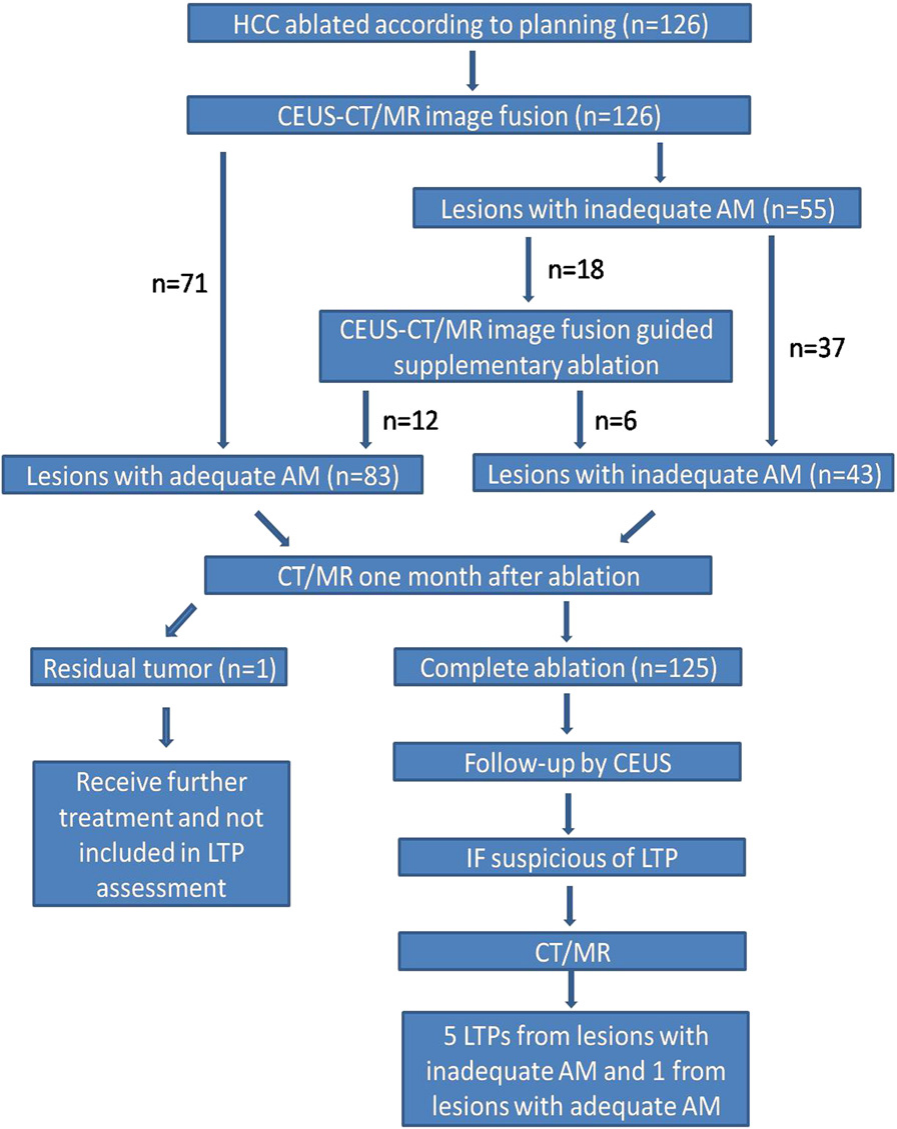
Flow diagram of intraoperative AM assessment by CEUS-CT/MR image fusion and management

**Fig. 2:**
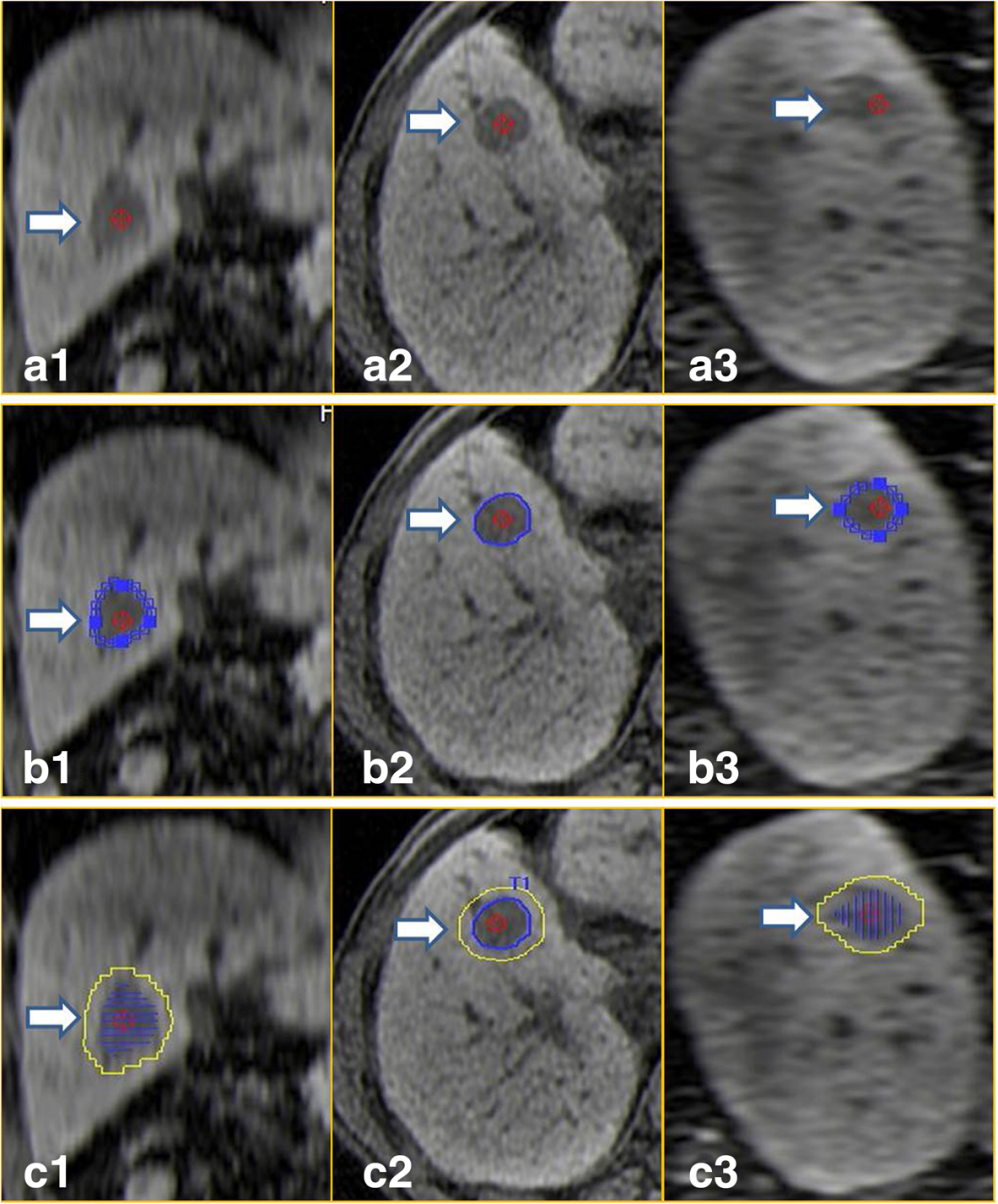
**a1**-**a3** One delayed phase series of MR in DICOM format was transferred into the navigation system, and the navigation system automatically generated the three-dimensional data and showed the transverse, coronal and sagittal plane of the tumor. **b1**-**b3** The index tumor is outlined in *blue*. **c1**-**c3** The 5-mm AM is outlined in *yellow*

A flow diagram of intraoperative AM assessment and management is shown in Fig. 1. The fusion was performed 10–15 min after ablation to decrease the interference of gas in the ablative area. First, one CT/MR portal or delayed phase series in DICOM format was transferred into the navigation system in MyLab Twice, which could be performed before ablation. The navigation system automatically generated the three-dimensional (3D) data and displayed the reconstructed 3D-CT/MR images. The index tumor and 5-mm AM were outlined in different colors (Fig. 2). For co-registration, the axial section of the medial line of the body between the CT/MR and the primary ultrasound scan was used. Vascular structures, such as bifurcations or confluences of the portal and hepatic veins, were frequently chosen as anatomical landmarks. After planar registration, additional refinement was performed to enable more precise fusion. All co-registrations and refinements were performed at the end of expiration, which was controlled by a breathing machine. Image fusion was only achieved at the area around the index lesion rather than the whole liver.

After ultrasound-CT/MR image fusion, CEUS was performed in the overlapped mode of CT/MR with CEUS. The patient’s breath was stopped at the end of expiration, and during the arterial, portal and delayed phase, the probe was moved across the entire ablative area to determine whether the non-enhanced ablative area encompassed the tumor as well as the AM. The CEUS-CT/MR image fusion images and clips were stored on a disk.

The time used to generate the CEUS-CT/MR image fusion (CT/MR data upload to the US machine not included) and the success rate of CEUS-CT/MR image fusion were recorded. The CEUS image quality and the CEUS-CT/MR image fusion for evaluating the AM were assessed intraoperatively by 2 independent assessors, who then conferred and arrived at a consensus. The CEUS image quality was classified as “good”, “medium” or “poor”. A CEUS image of an ablative area of good quality was defined based on the presence of clear and sharp margins. If the margin of the ablative area in the CEUS image could not be clearly visualized, it was defined as “poor”. An image quality between “good” and “poor” was defined as “medium”.

If the CEUS-CT/MR image fusion showed that the ablative area covered the whole tumor, it was defined as complete ablation. If the CEUS-CT/MR image fusion showed that the ablative area covered the whole AM, it was defined as adequate AM. If the ablative area just covered the whole tumor but not the AM, it was defined as an inadequate AM. The space around the index tumor was equally divided into 8 quadrants, designated 1–8, by three orthogonal transverse, coronal and sagittal planes crossing the center of the tumor. The occurrence of an adequate AM and the quadrant containing an inadequate AM were recorded. The reasons for an inadequate AM were categorized as blood vessel-related (diameter larger than 2 mm) and non-vessel-related. If the blood vessel was within 5 mm of the lesion and remained intact after ablation, it was considered blood vessel-related; otherwise, it was designated as non-vessel-related. After supplementary ablation, another CEUS-CT/MR image fusion was performed.

### Follow-up

One month after ablation, all patients received contrast-enhanced CT/MR as the standard for complete ablation. Complications related to ablation were also recorded. Patients with residual tumors would receive further treatment, and their patient data would not be used for the LTP evaluation. For patients with complete ablation, follow-up was performed using CEUS and serum AFP examination at 2-month intervals and CT/MR at 6-month intervals. If LTP was suspected by CEUS, CT/MR was performed. The imaging criteria for LTP on CT/MR included the presence of a characteristic enhancement pattern (hypervascularization in the arterial phase and a wash-out pattern in the portal and delayed phases) adjacent to the ablative area. The complete ablation rate and the occurrence and location of LTP were recorded.

### Statistical analysis

Univariate analysis and multivariate analysis were performed. For univariate analysis, we divided all of the tumors into 2 groups according to each variable that could potentially be related to LTP: (1) age (<70 years or >70 years), (2) sex (male or female), (3) etiology (hepatitis B/C positive or negative), (4) liver cirrhosis (yes or no), (5) Child–Pugh grade (A or B), (6) AFP (<20 ng/ml or >20 ng/ml), (7) number of tumors at the time of RFA (1 or >1), (8) tumor diameter (<20 mm or ≥20 mm), (9) past history of HCC (yes or no), (10) located in the hepatic dome (defined as within 10 mm beneath the diaphragm) (yes or no), (11) TACE before ablation (yes or no), and (12) AM (adequate or inadequate). The cumulative LTP rates of these 2 groups for each factor were estimated using the Kaplan–Meier method, and the statistical significance was assessed using the log–rank test. Next, multivariate analysis using the step-wise Cox proportional hazard model was performed for the variables with *P* < 0.20 in the univariate analysis to investigate independent risk factors for LTP.

The statistical analysis was performed using SPSS for Microsoft Windows (version 11.0.1; SPSS Inc. Chicago, IL, USA). Measurement data are presented as the mean ± standard deviation or the median. Counted data are expressed as cases or lesion numbers and percentages. *P* < 0.05 was considered statistically significant.

## Results

Ninety-eight patients with 126 HCC lesions were enrolled in the analysis. The clinical characteristics of the patients and lesions are listed in Table 1. Ultrasound and CT/MR images could not be successfully fused in 3 patients with 5 HCC lesions because of anatomical deformation, and these 3 patients were excluded. The rate of successful image fusion was 96.2 % (126/131), and the duration required for image fusion (time for transferring CT/MR image data into the ultrasound machine was not included) was 4.9 ± 2.0 (3–13) min. Artificial ascites and pleural effusion were used in 16 and 6 patients, respectively, and the median volume of normal saline used was 600 ml (400–1000 ml) and 400 ml (300–600 ml), respectively. Image fusion was successful in all lesions with artificial ascites or pleural effusion. Altogether, 147 contrast agent injections were applied, and the CEUS image quality was good in 36.1 % (53/147) and medium in 63.9 % (94/147) of the cases.

**Table 1:** Clinical characteristics of the patients and HCC lesions

Characteristics	Value
Gender (M/F)	91/7
Age (mean, range, years)	53.7 ± 10.5 (29 ~ 75)
Viral hepatitis/alcoholic liver disease	97/1
Liver cirrhosis (yes/no)	82/16
Child-Pugh class (A/B)	89/9
AFP (ng/ml) (<20/≥20)	65/33
History of HCC (yes/no)	50/48
Tumor number (1/>1)	75/23
Tumor diameter (mean, range, mm)	19.6 ± 7.7 (10 ~ 52)
Tumor diameter distribution (mm) (10–19/20–29/30–39/40–49/≥50)	69/40/15/1/1
RFA plus TACE (yes/no)	41/85

**Fig. 3:**
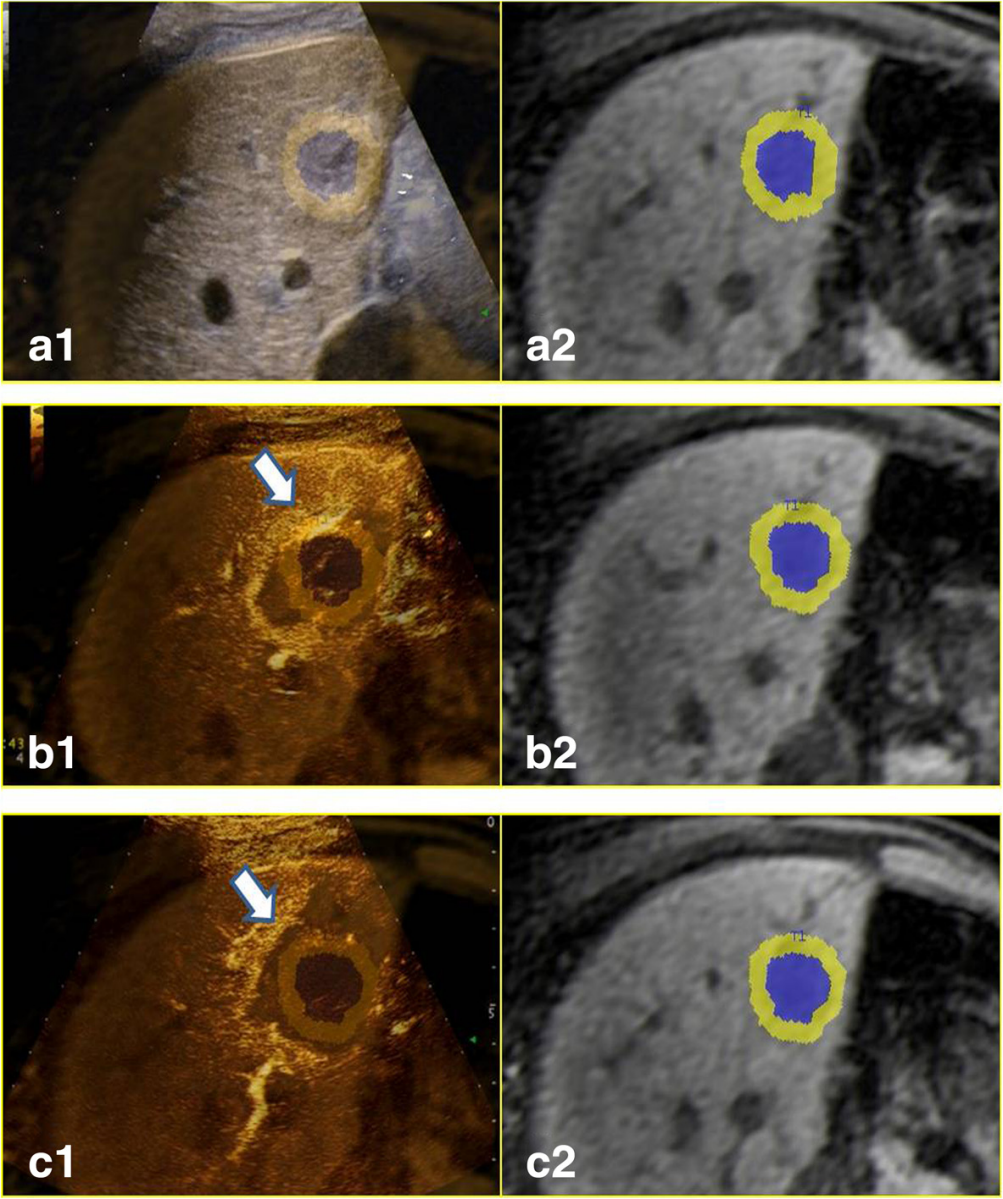
**a1** and **a2** For the lesion in Fig. 2, ultrasound image was fused with the MR image; a1 shows the overlapped ultrasound and MR image with the tumor in blue and the AM in yellow, and a2 shows the MR image with the tumor outlined and the AM. After ablation, CEUS-MR image fusion showed an inadequate AM (*arrow* in **b1**); **b2** shows the MR image with the tumor outlined and the AM. After supplementary ablation, the inadequate AM became an adequate AM (*arrow* in **c1**); **c2** shows the MR image with the tumor outlined and the AM

After ablation according to the predetermined plan, CEUS-CT/MR image fusion detected 55 lesions that did not achieve adequate AMs in 208 quadrants. Inadequate AMs were caused by blood vessels in 142 (68.3 %) quadrants and by non-vessels in 66 (31.7 %) quadrants. Supplementary ablation was applied in 18 lesions. In 12 of the 18 lesions, an inadequate AM in all quadrants resulted from non-vessel-related causes. In the other 6 lesions, inadequate AM in some quadrants resulted from non-vessel-related causes and in some other quadrants from blood vessel-related causes. In the 18 lesions, supplementary ablation was only applied to 44 quadrants with an inadequate AM due to non-vessel-related causes. After supplementary ablation, the inadequate AMs in the 44 quadrants became adequate AMs (Fig. 3). Because of supplementary ablation, 21.8 % (12/55) of the lesions with inadequate AMs achieved adequate AMs and 21.2 % (44/208) of the quadrants with inadequate AMs achieved adequate AMs. Finally, 43 lesions that did not achieve adequate AMs in 164 quadrants, including 142 quadrants (86.6 %) for blood vessel-related reasons and 22 quadrants (13.4 %) for non-vessel-related reasons (Table 2).

**Table 2:** Main reasons for inadequate AMs in quadrants

Reasons for inadequate AM	Number of quadrants
Blood vessel-related	142 (86.6 %)
Non-vessel-related	
Close to critical structures, supplementary ablation was not applied	14 (8.5 %)
Insufficient liver function, supplementary ablation was not applied	4 (2.4 %)
Tough puncture pathway, supplementary ablation was not applied	4 (2.4 %)
Total	164

One month after ablation, all patients received CT/MR, with 1 residue tumor detected. The success rate of CEUS-CT/MR image fusion for detecting complete ablation was 99.2 % (125/126). The lesion that was not completely ablated had indistinct boundaries and was assessed as having an adequate AM by CEUS-CT/MR image fusion. This patient later received TACE and was not included in further analyses in the present study. There was only 1 patient with a hemothorax after ablation, and catheterization was performed. The catheter was removed 6 days later, and the patient recovered.

**Fig. 4:**
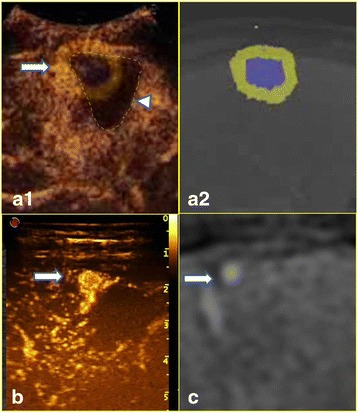
**a1** and **a2** show the CEUS-CT fused image. **a1** shows the overlapped image of CEUS and CT. The HCC lesion and 5-mm AM are outlined in *blue* and *yellow*, respectively. The ablative area in CEUS was anechoic and is outlined with a dotted line (*arrowhead*). In **a1**, the ablative area just covers the tumor, and part of the AM is not covered by the ablative area (*arrow*). Thus, the CEUS-CT fused image shows that this HCC lesion has an inadequate AM at the site indicated by the *arrow*. In the CT image in **a2**, the HCC lesion and 5-mm AM are outlined in *blue* and *yellow*, respectively. One month after ablation, the CT revealed complete ablation of the lesion, and the serum AFP fell to a normal level. However, 9 months after ablation, the serum AFP again increased. LTP was shown by CEUS (**b**) and CT (**c**) at the same site with an inadequate AM as in **a1** (*arrow*)

The median follow-up periods for lesions with adequate AMs and inadequate AMs were 23 months (6–37 months) and 25 months (4–37 months), respectively, and there was no significant difference between the follow-up periods of the 2 groups (*p* = 0.778). During follow-up, 6 LTPs were detected, and the rate of LTP was 4.8 % (6/125). One LTP occurred in a lesion with an adequate AM at 6 months after ablation, and the rate of LTP in this group was 1.2 % (1/82). This patient concomitantly presented multiple intrahepatic occurrences and subsequently received TACE. The other 5 LTPs occurred in lesions with inadequate AMs at 4, 6, 9, 10 and 12 months after ablation, and the rate of LTP in this group was 11.6 % (5/43). The rate of LTP in inadequate AM quadrants due to blood vessel-related and non-vessel-related causes were 2.1 % (3/142) and 9.1 % (2/22), respectively, and this difference was not significant (*p* = 0.134). The positions of LTP matched the positions of inadequate AMs, as detected by CEUS-CT/MR image fusion (Fig. 4).

In the univariate analysis, the potential factors contributing to LTP with *p* < 0.20 were liver cirrhosis (*p* = 0.150), tumor diameter (*p* = 0.118) and AM (*p* = 0.014) (Table 3). The multivariate analysis showed that the only independent risk factor for LTP was AM (hazard ratio, 9.167; 95 % confidence interval, 1.070-78.571; *p* = 0.043).

**Table 3:** Univariate analysis of possible risk factors for local tumor progression

Variables	Number	*P*-value
Age (<70 years/≥70 years)	117/9	0.508*
Sex (male/female)	114/12	0.464*
HBV/HCV infection (yes/no)	125/1	0.794*
Liver cirrhosis (yes/no)	105/21	0.150*
Child–Pugh grade (A/B)	113/13	0.715*
AFP level (<20 ng/ml/≥20 ng/ml)	69/57	0.699*
Number of tumors (1 or ≥ 1)	75/51	0.764*
Tumor diameter (<30 mm/≥30 mm)	109/17	0.118*
Past treatment history for HCC (yes/no)	58/68	0.496*
Located in the hepatic dome (yes/no)	39/87	0.848*
TACE before ablation (yes/no)	41/85	0.356*
Ablative margin (inadequate/adequate)	83/43	0.014*

*Log–rank test

## Discussion

The results of the present study support the feasibility of the clinical use of CEUS-CT/MR image fusion for evaluating AM intraoperatively after HCC ablation based on several features. First, image fusion between ultrasound and CT/MR could be achieved within 5 mins, which would not obviously prolong the operational time. Of course, the performance of this technique would require a learning curve. Based on our experience, approximately 30 practice repetitions would greatly shorten the registration time.

Second, a high success rate was obtained for CEUS-CT/MR image fusion. In previous studies, 3DCEUS-CT/MR image fusion [13] had a relatively low technical success rate (81.6 %), whereas in the present study, a much higher rate of successful image fusion was achieved (96.2 %). This difference might be explained by an ability of 2-dimensional CEUS-CT/MR image fusion to reduce the influence of narrow intercostal spaces or the location of the lesion [13]. Patients who were scheduled to receive liver resection, laparoscopic cholecystectomy or splenectomy were excluded from the analysis because these procedures would lower the successful rate of image fusion. In our study, we maintained accurate image fusion around the index lesion rather than the whole liver, so that anatomical changes caused by artificial ascites or pleural effusion would not affect the image fusion. Additionally, the breath of the patient was well controlled using a breathing machine for as long as 2 min, which would greatly facilitate co-registration.

Third, the image quality of the intraoperative CEUS was sufficient for further evaluation. The gas caused by ablation would affect the image quality of CEUS; consequently, we waited 10–15 min until the hyperechogenicity around the ablative area decreased. Attentive observation of the echogenicity caused by gas and microbubbles might improve interpretation of the CEUS image. The echogenicity caused by gas was present before arrival of the contrast agent and was not displaced. In contrast, the chogenicity caused by microbubbles was displayed due to their presence in the blood vessels.

In clinical cases, the AM is not verifiable by pathology; therefore, the LTP rate has been chosen to test the accuracy of the evaluated AM [10, 11, 13]. In the present study, the AM was the only independent factor affecting LTP, which indicated that CEUS-CT/MR image fusion provided an accurate evaluation of the AM. Compared with other studies [11, 12], a much lower LTP rate (4.8 %) was obtained in the present analysis. This difference might be explained by the much higher rate of adequate 5-mm AMs in this study compared with other studies. The high rate of adequate AMs resulted in part from intraoperative supplementary ablation, such that approximately 20 % of the inadequate AMs were effectively reduced. The other reason for the high rate of adequate AMs was that effort was made to ablate the tumor as well as the 5-mm AM. In the previous studies [11, 12] the authors did not describe their efforts to ablate the AMs. We also tried to ablate the vessel near the lesion if its diameter was less than 2 mm, which reduced the rate of inadequate AMs due to blood vessel-related causes.

In the case of an inadequate AM due to non-vessel-related causes, CEUS-CT/MR image fusion was more useful because it could not only detect the inadequate AM but also guide supplementary ablation. All of the inadequate AMs resulting from non-vessel-related causes were transformed into adequate AM after supplementary ablation, indicating that these types of AMs could be improved. For lesions in which supplementary ablation was not applied due to the presence of nearby critical structures such as the gallbladder and gastrointestinal tract, ethanol injection could be applied at the risky location.

Blood vessels near the lesion were the most common reason for an inadequate AM in our study, which is in agreement with previous studies [11]. Although no significant differences were observed, the rate of LTP related to inadequate AMs due to non-vessel-related causes (9.1 %, 2/22) was higher than that due to blood vessel-related causes (2.1 %, 3/142). This finding might be because, if the lesion was within 5 mm of a blood vessel, we attempted to ablate all of the normal hepatic parenchyma between the lesion and the vessel. This procedure could reduce the heat sink effect caused by blood flow, and micro-satellite nodules around the original tumor might also be treated, in turn lowering the rate of LTP [24, 25].

CEUS-CT/MR image fusion could also be used to evaluate complete ablation by observing whether the ablative area covered the entire tumor. The comparison of HCC before ablation and the ablative area after ablation was more objective than routine CEUS and more suitable for less experienced operators. This finding might explain why CEUS-CT/MR image fusion demonstrated a higher rate of detecting complete ablation (99.2 %) than routine CEUS (96.6 %) [15]. One residual lesion was recorded as an adequate AM. This lesion had an indistinct boundary, and it is possible that the area of the lesion had been underestimated. Whether CEUS-CT/MR image fusion could eventually improve the complete ablation rate requires further study.

This study had some limitations. First, the results of CEUS-CT/MR image fusion could only show whether the necrotic area covered the 5-mm thickness of the normal liver parenchyma around the HCC lesion. It could not offer biological evidence for the absence of viable tumor cells at the margin. Second, the difference in the occurrence of LTP occurrence was the only evaluating indicator, which might partially explain why a lesion with an adequate AM developed LTP. Theoretically, the type of tumor differentiation and the results of TACE before ablation might also influence LTP. A well designed clinical study or experimental study is needed to further evaluate CEUS-CT/MR image fusion for assessing AM. Third, supplementary ablation could potentially increase the rate of complications. Thus, future studies are needed to assess whether the AM should be covered in all lesion types to decrease LTP, such as lesions with a complete pseudocapsule.

## Conclusions

In conclusion, these present findings demonstrated the feasibility of intraoperative evaluation of the AM using CEUS-CT/MR image fusion after HCC ablation. Supplementary ablation could be guided by CEUS-CT/MR image fusion, leading to improved AMs. The failure to establish a 5-mm AM was the only significant risk factor associated with concordant LTP, thus demonstrating the accuracy of CEUS-CT/MR image fusion for evaluating AMs. This study describes a new method to evaluate and improve AMs, which might help decrease the rate of LTP.

## Availability of supporting data

The datasets supporting the conclusions of this article are presented in the main manuscript.

## Abbreviations

HCC: hepatocellular carcinoma
CEUS: contrast-enhanced ultrasound
LTP: local tumor progression
RFA: radiofrequency ablation
AM: ablative margin
3D: three-dimensional
